# Comparison of Midazolam Plasma Concentrations in ARDS Patients with and Without ECMO Support—Prospective Observational Pilot Study

**DOI:** 10.3390/life16071144

**Published:** 2026-07-10

**Authors:** Marek Grochla, Marcin Basiak, Bogusław Okopień, Ewa Trejnowska, Piotr Knapik

**Affiliations:** 1 Department of Anesthesiology and Intensive Therapy in Zabrze, Medical University of Silesia in Katowice, Ul. Marii Skłodowskiej-Curie 9, 41-800 Zabrze, Poland; etrejnowska@sum.edu.pl (E.T.); pknapik@sum.edu.pl (P.K.); 2 Department of Internal Medicine and Clinical Pharmacology in Katowice, Medical University of Silesia in Katowice, Ul. Medyków 14, 40-752 Katowice, Poland; mbasiak@sum.edu.pl (M.B.); bokopien@sum.edu.pl (B.O.)

**Keywords:** midazolam, drug monitoring, ARDS, ICU, ECMO

## Abstract

Introduction: Patients undergoing ECMO therapy have a much higher need for drug therapy due to the increased volume of distribution and deeper levels of sedation. The influence of ECMO support on the achieved midazolam concentrations is unknown. Materials and Methods: This prospective, single-center study was conducted between October 2022 and December 2024. All mechanically ventilated patients with an FiO_2_ over 0.6 or requiring VV ECMO for respiratory support were included. Patients younger than 18 years, patients without midazolam infusion, and patients with a do-not-resuscitate protocol were excluded. Patients were divided into a group requiring ECMO therapy (group A, n = 14, 88 measurements) and a group receiving conventional respiratory support (group B, n = 11, 44 measurements). Mean daily doses of midazolam and achieved midazolam concentrations were compared. Chi-squared tests, Mann–Whitney U tests, Pearson’s r correlation, and Spearman’s rank correlation were used to assess statistical significance between groups, where appropriate. Results: In terms of demographic data, patients in group A were comparable to group B, except that patients in group A had a lower BMI (25.7 ± 5.1 kg/m^2^ vs. 34.0 ± 10.1 kg/m^2^, *p* = 0.014). Midazolam requirements in the ECMO group were higher (3.6 ± 1.6 mg/kg vs. 1.8 ± 1.3 mg/kg, *p* < 0.001). Despite this, midazolam plasma concentrations in both groups were comparable, and only midazolam metabolites were found to be higher in group A (60 ± 72 ug/L vs. 22 ± 19 ug/L; *p* < 0.001). Conclusions: The use of probably higher doses of midazolam does not translate into higher plasma midazolam concentrations in ECMO patients.

## 1. Introduction

Benzodiazepines constitute a class of medications frequently administered in intensive care units (ICUs) to achieve pharmacologically induced sedation. Among the most widely used agents in this group is midazolam, a gamma-aminobutyric acid (GABA) receptor agonist [1,2,3].

Single doses of midazolam administered during anesthesia are generally safe when given by experienced personnel. However, the situation differs in the ICU setting, where substantially higher doses are often required and administered over prolonged periods. Furthermore, numerous drug interactions may occur in critically ill patients.

Benzodiazepines induce amnesia, which is considered a desirable effect in ICU patients, as hospitalization in these units is often associated with significant psychological stress and trauma. However, prolonged administration may lead to the development of tolerance, necessitating increasing doses to achieve the same sedative effect. In addition, benzodiazepine dependence may develop, making the reduction and discontinuation of midazolam a prolonged and challenging process [2,3,4,5].

The bispectral index (BIS) is commonly used to assess the depth of sedation. This neuromonitoring technique is based on electroencephalography (EEG) and evaluates the brain’s bioelectrical activity. BIS values range from 0 to 100, where 0 indicates the absence of cerebral electrical activity and 100 corresponds to a fully awake state. BIS monitoring is non-invasive and safe, with no absolute contraindications to its use. However, its accuracy may be affected by interference from other electrical devices. Notably, ketamine is the only commonly used sedative agent for which BIS monitoring is considered unreliable [5,6,7,8].

Patients undergoing extracorporeal membrane oxygenation (ECMO) therapy often require substantially higher doses of sedative agents due to an increased volume of distribution and drug sequestration within the ECMO circuit, particularly on the oxygenator membrane [9,10]. In addition, ECMO therapy may be associated with cytochrome P450 activation, resulting in enhanced midazolam metabolism [11]. Patients receiving ECMO support almost invariably meet the criteria for polypharmacy, commonly defined as the concurrent use of more than five medications. Consequently, numerous drug–drug interactions may occur, many of which are difficult to avoid in the ICU setting. Among the most clinically relevant adverse effects are QT interval prolongation and bleeding complications, with the latter largely related to the use of systemic anticoagulation.

In our study, we aimed to answer the following questions:

- Do patients receiving ECMO therapy require significantly higher doses of sedatives to maintain a similar state of sedation compared with other ARDS patients?
- What is the influence of ECMO on achieved midazolam concentrations?
- Do ECMO patients receive a midazolam toxic dose?
- What is the difference between patients with and without ECMO?
- Do ECMO patients have lower mortality rates?

## 2. Materials and Methods

This prospective single-center study was carried out in the intensive care unit (ICU) of the university hospital affiliated with the Medical University of Silesia in Katowice, Poland. This study was performed between October 2022 and December 2024. All mechanically ventilated patients with an FiO_2_ over 0.6 or requiring VV ECMO for respiratory support were included. The objective of the research was to juxtapose the group of patients with ARDS requiring VV ECMO (group A) and group of patients with ARDS not requiring VV ECMO (group B).

The study excluded patients younger than 18 years, patients without midazolam infusion, patients with a do-not-resuscitate protocol, and patients not requiring mechanical ventilation. The research was approved by the Bioethics Committee of the Medical University of Silesia in Katowice—PCN/022/KB1/55/II/21/22 from 18 October 2022. For the objective of this research, the Ethical Committee of the Medical University of Silesia allowed for a proxy consent from direct members of family (spouse, parents, or children).

All qualified patients were examined twice daily to assess their sedation level according to the RASS (Richmond Agitation–Sedation Scale) scale. The score on this scale ranges from −5 (non-reactive patient) to +4 (agitated, delirious patient). The RASS score and the BIS findings were subsequently analyzed for correlation with plasma midazolam concentrations. Kidney and liver function tests were also performed on days when blood samples were taken to assess midazolam plasma levels. Kidney function was assessed using the Cockcroft–Gault pattern, while liver function was assessed with the Model of End-Stage Liver Disease (MELD)—scoring from 6 to 40 points. On days when midazolam was administered, the daily dose of midazolam was assessed. This was later correlated with the plasma levels of midazolam.

BIS monitoring was provided using the Covidien Medtronic BIS^TM^ monitoring system (Galway, Ireland) and the dedicated BIS Quatro Sensor (Galway, Ireland). The BIS test and RASS were conducted twice daily (9:00 and 21:00). Kidney and liver function tests (INR, total bilirubin, and creatinine) and lactate levels were performed on the same days that blood samples were collected for plasma midazolam levels.

Midazolam plasma concentrations were collected twice weekly at 9:00. Patients’ plasma samples were transferred from a volume of 50 μL to 1.5 mL Eppendorf reaction tubes and prepared in accordance with the producer’s protocol for the reagent kit used to assess benzodiazepines by liquid chromatography–tandem mass spectrometry (LC-MS/MS) with the ClinMass^®^ TDM Kit System Benzodiazepines in Serum/Plasma (RECIPE Chemicals, Munich, Germany). Prepared samples were placed in an Agilent 1290 Infinity II liquid chromatograph autosampler (Santa Clara, CA, USA). Chromatographic separation was performed with a sample injection volume of 2 μL using the analytical column and mobile phases provided in the reagent kit from the manufacturer’s recipe. The temperature of the chromatography oven was programmed at a temperature of 40 °C, and the flow rate of the mobile phase was maintained at 0.6 mL/min in an alternating two-phase gradient according to the documentation provided by the manufacturer. The total separation time for one sample was 7.3 min. The outlet of the chromatography column was directly coupled to an ion source functioning in positive electrospray ionization (ESI) mode of the Sciex Triple Quad 3500 mass spectrometer (SCIEX, Marlborough, MA, USA). The mass spectrometric analysis was conducted with the tandem mass spectrometer set to multiple reaction monitoring (MRM) mode.

Two fragmentation reactions were analyzed for each compound within the mass range provided by the reagent manufacturer. For each analyzed compound, the kit manufacturer provided a radiolabeled internal standard (ISTD). Sciex Analyst and Sciex Multiquant 1.7.3 software (SCIEX, Marlborough, MA, USA). were used to collect and process the data. Calibration was performed, consisting of three concentration points with the ClinCal^®^ Serum Calibrator Set lyophilized for benzodiazepines (RECIPE Chemicals, Munich, Germany, LOT 1069). Quality control of the performed markings was checked using the ClinChek^®^ Serum Control lyophilized for the benzodiazepines kit (RECIPE Chemicals, Munich, Germany, LOT 1267) by analyzing the control samples at two concentration levels.

Patients were classified as having “cardiac failure” when they had previously been diagnosed with cardiac failure, had a reduced left ventricular ejection fraction (<50%), or required the use of catecholamines (dobutamine or epinephrine) or mechanical circulatory support or had pulmonary hypertension. Patients with previously diagnosed renal injury or patients who required renal replacement therapy (RRT) were classified as having “renal failure”. Patients were classified as having “liver failure” if they had a diagnosis of liver failure (or signs of liver failure such as prolonged INR (>3x times) and increased total bilirubin concentrations (>2x times)) or needed SPAD or Cytosorb.

Scale variables are shown as the standard deviation and mean, whereas categorical parameters are presented as percent. Pearson’s r correlation, Mann–Whitney U tests, Spearman’s rank correlation, and Chi-squared tests were used to assess statistical significance between groups, where applicable. Laboratory parameters and distinct demographic data were compared between patients receiving ECMO and patients not receiving ECMO. The result of distinct variables on mortality was counted using univariable logistic regression. The authors of the study considered a two-tailed *p*-value < 0.05 statistically significant for all conducted analyses. The graphs and analyses were prepared using Dell Statistica (version 13) software.

## 3. Results

Overall, 25 patients were analyzed in this study—14 in group A (V-V ECMO) and 11 in group B (patients not requiring ECMO).

Comparison of demographic data revealed that patients in group A had a significantly lower body weight and thus a lower BMI (*p* = 0.014) and longer ICU stay (*p* = 0.017); however, most demographic data were comparable. Thiopental was used often by ECMO patients (*p* = 0.001). Preoperative mean APACHE II (Acute Physiology and Chronic Health Evaluation II) scores were similar in both groups. ECMO patients were characterized by a significantly longer ICU stay. Mortality in the analyzed groups was similar; however, this study was not designed to answer questions on the patients’ outcomes: Table 1 and Table 2.

All collected samples correspond to the cumulative dose administered over the 24 h preceding blood collection. Midazolam plasma concentrations in both groups were similar; however, midazolam metabolite plasma concentrations were higher in group A (252.4 ± 184.5 mg vs. 217.2 ± 166.0 mg, *p* = 0.368 and 60 ± 72 µg/L vs. 22 ± 19 µg/L; *p* < 0.001, respectively). There was also a significantly higher requirement for midazolam in group A, both in the direct comparison and after conversion into mg/kg (3.6 ± 1.6 mg/kg vs. 1.8 ± 1.3 mg/kg, *p* < 0.001). All of these results are presented in Table 3.

Our population had different etiologies causing ARDS. However, pneumonia and pulmonary fibrosis predominated. The rest of the results are published in Table 4.

**Figure 1:**
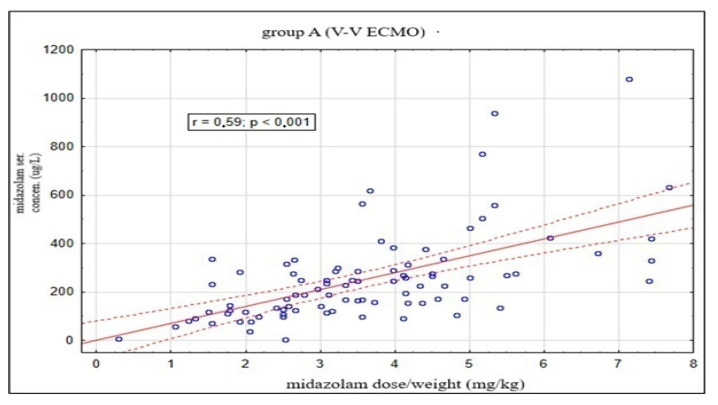
Correlation between midazolam plasma concentration and quotient midazolam dose/weight with ECMO patients.

**Figure 2:**
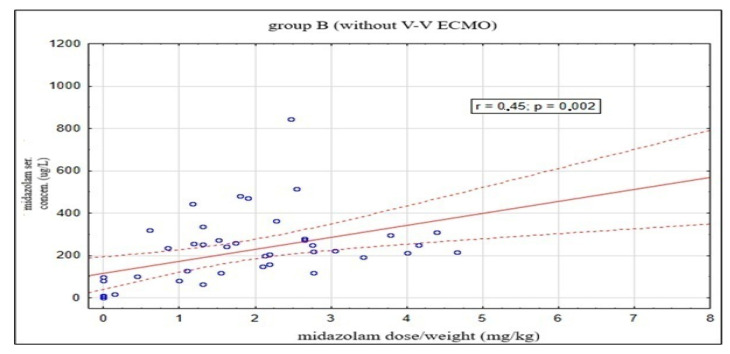
Correlation between midazolam plasma concentration and quotient midazolam dose/weight without ECMO patients.

There is a positive correlation between midazolam concentration and the midazolam dose per kg in both study groups. The correlation for the ECMO group is r = 0.59; *p* < 0.001, and for the non-ECMO group, it is r = 0.45; *p* = 0.002. The results are presented below in Figure 1 and Figure 2.

## 4. Discussion

Benzodiazepines represent the primary class of agents used to achieve sedation in critically ill patients [2]. In our study, patients receiving ECMO support required significantly higher doses of midazolam than those who did not undergo ECMO therapy. On the other hand, this may result from a greater need for midazolam sedation in patients who may react with excessive hypotension after the use of higher propofol flows. Significantly higher α-hydroxymidazolam (α-OH-midazolam) concentrations were also observed; however, no significant differences were found in plasma midazolam concentrations. These findings suggest enhanced drug elimination in patients receiving ECMO support. This phenomenon may be attributed to an increased glomerular filtration rate and a larger volume of distribution observed in this patient population. In our pilot study, patients receiving ECMO support exhibited higher α-hydroxymidazolam (α-OH-midazolam) concentrations. Several mechanisms may contribute to this finding. First, ECMO therapy is associated with an increased volume of distribution, which may alter the pharmacokinetics of sedative agents [12]. Second, the severity of critical illness may affect both hepatic and cardiac function, thereby influencing drug metabolism and clearance. In patients with renal dysfunction, the excretion of α-OH-midazolam, an active metabolite of midazolam, may be impaired, resulting in its accumulation [13].

Furthermore, patients with severe respiratory failure frequently develop dysfunction of other organ systems. Extracorporeal therapies, particularly ECMO, are also associated with increased hemolysis and elevated concentrations of free hemoglobin. Free hemoglobin is highly cytotoxic, and its accumulation may contribute to further organ injury, potentially affecting the pharmacokinetics and elimination of administered medications [12].

Previous studies have questioned the utility of routine midazolam plasma concentration monitoring in critically ill patients. For example, Canadian investigators concluded that routine measurement of midazolam concentrations is not warranted in all patients [2].

The relationship between clinical sedation scales, BIS values, and plasma midazolam concentrations remains controversial. In a German study, no significant correlation was observed between BIS measurements, Richmond Agitation–Sedation Scale (RASS) scores, and plasma midazolam concentrations. In contrast, Bremer et al., whose findings were subsequently cited by the same authors, reported a statistically significant correlation between these parameters [1].

Given the complications associated with prolonged sedative use, including tolerance and dependence, accurate assessment of sedation depth is essential. Both bispectral index (BIS) monitoring and Richmond Agitation–Sedation Scale (RASS) assessment remain valuable tools, providing information on the adequacy of sedation and the potential for patient arousal during nursing and therapeutic procedures [7,8,14].

To date, no comparable studies involving patients with COVID-19 undergoing ECMO support have been reported. However, several studies have investigated the pharmacokinetics of midazolam and its plasma concentrations in patients receiving ECMO therapy.

In a retrospective study by Verkerk et al., patients with obesity receiving ECMO support did not require higher doses of midazolam than non-obese patients undergoing ECMO therapy [15]. In another study published in 2017, which evaluated sedation practices in a cohort of 32 patients receiving either venovenous (VV) or venoarterial (VA) ECMO support, delirium developed in approximately 50% of patients. The mean daily midazolam dose administered during ECMO therapy was 24 mg [16]. In contrast, a study conducted in North America reported a substantially higher mean daily midazolam dose of 202 mg in patients receiving ECMO support [17].

Propofol infusion has also been compared with midazolam infusion during ECMO therapy with no observed differences in oxygenator durability [18]. In another study, the authors reported a significant reduction in propofol concentration (by 70%) and midazolam concentration (by 46%) within 30 min of ECMO initiation [19].

In the present study, venovenous (V-V) ECMO therapy was not associated with a significant reduction in mortality. This finding is likely attributable to the limited sample size. Similar results were reported in the EOLIA trial, one of the landmark randomized controlled studies evaluating ECMO in patients with severe acute respiratory distress syndrome (ARDS). In that study, the 60-day mortality rate was 35% in the ECMO group compared with 46% in the conventional treatment group. Although the absolute reduction in mortality was 11%, the difference did not reach statistical significance. Notably, 28% of patients initially assigned to the control group were crossed over to ECMO after a median of 4 days, which may have influenced the study outcomes [20].

Further randomized trials comparing ECMO with conventional management in patients who clearly meet ECMO eligibility criteria are unlikely to be conducted, as ECMO is increasingly regarded as a rescue therapy in cases of refractory respiratory failure, raising ethical concerns regarding the withholding of treatment [20].

The overall survival rate in our cohort was 56%, which is consistent with previously published data. In the EOLIA trial, survival was 59.5%, while reported survival rates in patients with severe ARDS generally range between 50% and 60% [20]. Furthermore, six-month survival in our study remained favorable at 86%.

In our study, only two patients died within 6 months (in the group of 14 patients who survived intensive care). The accessible literature presents similar results, with level of mortality in patients with ARDS happening primarily in the acute phase and lowering thereafter. Mortality ranges from 5% to 80% if the six-month period [21,22,23]. Undoubtably, intensive care and rehabilitation are needed for these patients, since they experience increased physical and mental impairment. Extended immobilization and sedation contribute to muscular decline [21,24,25].

Another objective of this study was to assess whether the administered midazolam doses could be considered potentially toxic. However, this question is difficult to answer conclusively. Isolated forensic reports have suggested that a lethal dose of midazolam may be approximately 6.7 mg/kg body weight [26]. Nevertheless, such data should be interpreted with caution, as critically ill patients in the ICU typically receive midazolam as a continuous infusion over a 24 h period rather than as a single bolus dose, as described in forensic investigations.

Moreover, high doses are generally administered only during periods when deep sedation is clinically required. In the present study, although some patients receiving V-V ECMO support received daily doses approaching 6.7 mg/kg on individual days, their mean daily dose was 3.6 mg/kg. Furthermore, in the forensic cases cited above, overdose-related mortality was primarily associated with respiratory depression and asphyxia. In contrast, ICU patients receiving continuous midazolam infusions are most often mechanically ventilated, which substantially modifies the clinical consequences of high-dose benzodiazepine administration.

Patients with severe respiratory failure frequently require deeper levels of sedation. Accordingly, even patients not receiving ECMO support required a mean midazolam dose of 1.8 mg/kg/day in our study. This value exceeds the dose range reported by Boudin et al., who described typical ICU midazolam requirements of 0.03–0.3 mg/kg [26]. Similar dosage ranges are also reported in the Material Safety Data Sheet for midazolam [13].

The principal limitations of this study are its single-center design and the relatively small sample size. The study was conceived at the peak of the COVID-19 pandemic in Poland (2020–2021) and was primarily focused on patients with severe COVID-19-related respiratory failure. During this period, a dedicated section of the ICU was reserved exclusively for the treatment of patients with COVID-19.

Following grant approval and acquisition of study materials, the incidence of COVID-19 decreased substantially. Concurrently, the number of patients requiring ECMO support and prolonged ICU treatment declined, resulting in a considerably smaller study population than originally anticipated.

The limited sample size and data structure represent important methodological constraints. Multiple repeated measurements of clinical and pharmacokinetic parameters, including RASS score, BIS values, PaO_2_/FiO_2_ ratio, midazolam dose, and plasma midazolam concentration, were collected from individual patients. However, the number of enrolled patients was insufficient to allow for the use of advanced statistical approaches for longitudinal data analysis, such as generalized estimating equations (GEEs) or mixed-effects models, which require larger sample sizes to provide stable and reliable estimates.

To reduce the risk of artificially inflating statistical significance due to repeated observations, the analyses were performed at the patient level by aggregating repeated measurements (e.g., using mean or median values for each patient). Consequently, the findings should be regarded as exploratory. In particular, the observed associations between midazolam dose, plasma concentration, and clinical outcomes should not be interpreted as evidence of causality.

Furthermore, owing to the limited sample size, multivariable analyses could not be performed. The reported associations may therefore be confounded by disease severity and other unmeasured factors. Larger prospective studies using appropriate longitudinal statistical models are warranted to validate these findings and further investigate the pharmacokinetics of midazolam in patients receiving ECMO support. Due to the lack of precise timing between the initiation of the midazolam infusion and sample collection, more detailed pharmacological analyses were not performed. Further research is needed regarding the use of adequate sedation by ECMO patients.

Midazolam monitoring may be affected by concomitant medications. Another limitation of the study is that BIS was performed in only 50% of patients due to device failure. A strength of the study is that all patients were enrolled by the first author, which may have reduced selection bias and improved consistency of data collection, in line with the prospective design. On the other hand, this has its limitations in the form of a lack of blinding during study recruitment. However, given the observational nature of this work, it should not have a significant impact on the results.

## 5. Conclusions

The use of probably higher doses of midazolam does not translate into higher plasma midazolam concentrations in ECMO patients. More research is needed to confirm TDM midazolam use by ECMO patients.

## Figures and Tables

**Table 1:** Comparison of demographic data for both groups (per number of patients).

	Group A	Group B	*p*
	V-V ECMO	Without ECMO	U M-W/
	(n = 14)	(n = 11)	Fisher
Age (years)	47.4 ± 14.6	50.0 ± 15.0	0.528
Height (cm)	175.7 ± 11.4	175.3 ± 4.5	0.681
Weight (kg)	79.6 ± 17.5	103.6 ± 27.8	**0.017**
BMI (kg/m^2^)	25.7 ± 5.1	34.0 ± 10.1	**0.014**
APACHE II (points)	22.3 ± 7.5	22.0 ± −5.2	0.660
SAPS III (points)	59.5 ± 12.6	53.2 ± 13.5	0.250
ICU stay (days)	40.6 ± 19.3	24.5 ± 21.9	**0.017**
ECMO using time (days)	29.9 ± 21.8		
Ventilation time (days)	36.1 ± 19.6	22.4 ± 17.0	**0.037**

SAPS III—The Simplified Acute Physiology Score III, APACHE II—Acute Physiology and Chronic Health Evaluation II, ICU—intensive care unit.

**Table 2:** Comparison of demographic data for both groups (per number of patients).

	Group A	Group B	*p*
	V-V ECMO	Without ECMO	U M-W/
	(n = 14)	(n = 11)	Fisher
Acute lung failure	11 (78.6%)	8 (72.7%)	0.898
Women	5 (35.7%)	4 (36.4%)	0.999
Kidney injury	11 (78.6%)	9 (81.8%)	0.763
Liver failure	8 (57.1%)	4 (36.4%)	0.529
Cardiac failure	10 (71.4%)	9 (81.8%)	0.895
COVID-19	0 (0%)	2 (18.2%)	0.357
Thiopental	13 (92.9%)	2 (18.2)	**0.001**
Propofol	14 (100%)	10 (90.9%)	0.902
Ketamine	14 (100%)	7 (63.6%)	0.056
Opioids	13 (92.9%)	7 (63.6%)	0.190
Dexmedetomidine	14 (100%)	7 (63.6%)	0.056
Neuromuscular blockade	11 (78.6%)	7 (63.6%)	0.706
CVVHD/CVVHDF	8 (57.1%)	8 (72.7%)	0.677
Death	6 (42.9%)	5 (45.5%)	0.999

CVVHDF—Continuous Venovenous Hemodiafiltration; CVVHD—Continuous Venovenous Hemodialysis.

**Table 3:** Comparison of variables for both groups (repeated measurements).

	Group A		Group B		*p*
	V-V ECMO		Without ECMO		U M-W
Midazolam. plasma conc. (µg/L)	n = 88	252.4 ± 184.5	n = 44	217.2 ± 166.0	0.368
α-OH-midazolam plasma conc. (µg/L)	n = 88	60 ± 72	n = 44	22 ± 19	**<0.001**
GFR (mL/min/1.73 m^2^)	n = 69	178.2 ± 79.0	n = 34	135.0 ± 57.0	**0.006**
MELD (points)	n = 80	10.8 ± 3.1	n = 31	11.2 ± 4.5	0.844
Midazolam dose (mg)	n = 87	287.6 ± 128.1	n = 44	188.6 ± 145.4	**<0.001**
Midazolam dose/weight (mg/kg)	n = 87	3.6 ± 1.6	n = 44	1.8 ± 1.3	**<0.001**
RASS (points)	n = 95	−3.9 ± 1.2	n = 40	−3.1 ± 2.1	**0.027**
BIS (points)	n = 69	54.5 ± 17.5	n = 10	62.3 ± 18.9	0.232
Plasma lactate (mmol/L)	n = 86	1.1 ± 0.5	n = 43	1.7 ± 1.8	0.056
Mean of samples	n = 88	6.2 (2–12)	n = 44	4 (2–8)	-

RASS—Richmond Agitation–Sedation Scale, BIS—bispectral index, MELD—Model of End-Stage Liver Disease.

**Table 4:** The causes of ARDS.

Diseases	Number	The Causes of Pneumonia	The Causes of Chronic Fibrosis
Pneumonia	8	COVID-19 (1)	Hypersensitivity pneumonitis (2)
Wegener’s granulomatosis	2	Influenza A (2)	Pneumoconiosis (1)
Chronic fibrosis	5	Streptococcus pyogenes (1)	Idiopathic (2)
Cardiogenic shock	4	Legionella (1)	
IPAH	1	RSV (1)	
Burn	1	Acinetobacter baumannii (1)	
Rest	4	Idiopathic (1)	

IPAH—Idiopathic Pulmonary Arterial Hypertension.

## Data Availability

The article contains data.

## Abbreviations

The following abbreviations are used in this manuscript:

ICU	Intensive Care Unit
GABA	Gamma-aminobutyric Acid
ARDS	Acute Respiratory Distress Syndrome
EEG	Electroencephalography
RASS	Richmond Agitation–Sedation Scale
CVVHD	Continuous Venovenous Hemodialysis
CVVHDF	Continuous Venovenous Hemodiafiltration
ECMO	Extracorporeal Membrane Oxygenation
NO	Nitric Oxide
APACHE II	Acute Physiology and Chronic Health Evaluation II
SAPS III	The Simplified Acute Physiology Score III
MELD	Model of End-Stage Liver Disease
BIS	Bispectral Index
